# Diphtheria of the Ear

**Published:** 1887-12

**Authors:** Robert Barclay


### ABSTRACTS AND EXTRACTS.

### DIPHTHERIA OF THE EAR.

BY ROBERT BARCLAY, M. D.

[A paper read before the St. Louis Medical Society.]

The subject of diphtheria, which has of late been receiving your earnest attention, and which as a throat disease has, in a recent address, been so ably discussed, deserves a broader consideration by you as general practitioners of medicine.

Dr. H. C. Moir, in his "Manual of the Practice of Medicine," which is a careful résumé of the standard works of Niemeyer, Roberts, Loomis, DaCosta, Bristow, Hartshorn, and others, says: "It can not be well denied that it [diphtheria] is, first, a constitutional, and, secondly, a local disease; for it seems proven there is a local manifestation of a constitutional development by the fact that the disease can not be destroyed by taking off the various layers of the mucous membrane. It is unquestionably a constitutional disease, and the local changes depend entirely upon the amount of infection."

Intelligent treatment will depend mainly upon a correct determination of the mooted question, whether diphtheria is a constitutional disease primarily, and local secondarily, or *vice versa*. This solution will prove less difficult by making a broader study of diphtheria than is possible in the literature and observation of diphtheria of the pharyngo-larynx alone.

In searching broadly I find numerous reports of diphtheria affecting the genital organs, the eye, wounds, etc. Since the careful study of diphtheria of the eye which was made in 1884 by Dr. A. von Graefe, ophthalmologists have made further observations, numerous and careful. The frequent invasion of the organ of hearing by diphtheria has long since attracted the attention of otologists; to them the destructive features of this disease upon the ear are familiar, being seen so frequently in neglected aural cases which have been prematurely pronounced "cured" of their diphtheria.

In the excellent recent address upon the "local"—*i. e.*, the faucal and nasal —treatment of diphtheria, owing to its author's having been unfortunately misled in his search for the literature of diphtheria of the ear, the opinion was hazarded that the ear was but rarely attacked by this disease. Lest this should mislead you into carelessness in noting the condition of the organ of hearing, and, through ignorance of the truth, into neglect to protect from invasion of these parts the patients of diphtheria who may now or hereafter be under your professional care, you are here offered a statement of the facts, together with a brief rèsumè of the American and European literature concerning diphtheria of the ear, so far as I have found time and opportunity to consult it.

Passing over the cases which in the recent address are said to have been reported by Urbantschitsch, I give you the following:

Dr. S. Moos publishes a full history of a case of idiopathic diphtheria of the external auditory meatus. As this case is so often referred to by writers on the subject before us, and as it is so typical, I append an abstract of that history, as follows:

A boy ten years old had been twice under treatment by Dr. Moos for purulent otitis media of the left side—first, from July 24 till November 20, 1864, when he was discharged cured and with the perforation closed; and, secondly, after an attack of measles, from January 29 till March 19, 1865, when he was discharged cured of otorrhœa, but with persistent perforation. On August 18th he returned to Dr. Moos, *having suffered intense, constant and increasing earache for thirty-six hours preceding, with fever, loss of appetite, excessive thirst, and, during the second night, delirium.* Deglutition was easy, but mastication impossible because of its painfulness; patient pale and exhausted; pulse weak and 108 to the minute; furred tongue, and *the palate, tonsils, etc., were clean;* traction on auricle, and pressure on tragus painful; glands near by swollen and tender; inner surface of concha bright red; posterior surface of tragus, regio intertragica, and whole surface about outer end of meatus—whose inner portion could not be examined because of contraction—was covered with what is described as "a rather thick, inflexible, lardaceous, greasy coating," which could not be raised from the underlying structure; attempted separation resulted in only lacerating the parts matted together, with bleeding, and gave the patient intense pain; there was almost deafness on the affected side, except by bone-conduction; and *though the ear emitted a very offensive odor, there was no positive otorrhœa.*

Treatment consists in daily penciling with solution of nitrate of silver (gr. xv. to unc. j.) and frequently washing the parts with warm water. Cold applications of Goulard's solution (1 to 3), to relieve pain, with good effect. Mastication being painful, fluid food and broths only were given. Local phlebitis was contra-indicated by the poorly nourished condition of the patient. There was still fever, loss of appetite, and great restlessness at night.

August 26th and 27th, insomnia at night. On August 27th, coating about the tragus and entrance to the meatus began to peel off, accompanied by slight suppuration and considerable hæmorrhage.

August 29th, exfoliation began in meatus, accompanied by increased hæmorrhage. Syringing produced considerable hæmorrhage, and for the first time there was a decrease of the pain.

September 2d and 3d, repeated hæmorrhage; no pain. On September 3d, no fever, increased appetite; hearing distance for watch, $\frac{5}{72}$; for voice, eight paces; slight tenderness of ear on traction or pressure. Spots on outside of tragus, etc., described above, now show a sharply marked edge, with slightly granulating and suppurating surface; external meatus presented same appearance; swelling decreased, and, after syringing, speculum was easily introduced, through which old perforation of drumhead was plainly recognized; the drumhead itself was seen to be covered with grayish-red pus, but itself not clearly made out. Improvement followed in external meatus, but a relapse of the otitis media prolonged treatment until October 22d, when patient was discharged cured of his disease, but with a persistent perforation, as before.

Dr. Moos states that "Wreden has observed five cases of diphtheria of the external ear, and in those most carefully noted the membrana tympana was implicated."

Dr. Adam Politzer writes of croupous otitis externa as a rare form of disease of the external meatus, and refers to cases reported by Dr. William R. Wilde; also to a case, reported by Dr. Gottstein, of croupous exudation of the tonsils and posterior wall of the osseous auditory meatus.

Dr. Bezold reports eleven cases of this affection met with in three years.

Dr. Politzer says that otitis externa diphtheritica is seldom primary, but it is usually a complication of scarlatinal diphtheria of the throat and middle ear. But primary diphtheria of the meatus has been observed and reported by Drs. Moos, Bezold, Wreden, Callan, and Kraussold, which developed during an epidemic of diphtheria, but upon a previously existing otitis externa or excoriated condition of the meatus.

Dr. Blau mentions the necrosis and shedding of the membrane, and reports a case of diphtheria of the external and middle ear simultaneously.

Dr. Jacobson reports three cases of diphtheria of the canal observed at Dr. Lucae's clinic.

Dr. Bezold reports three cases.

Dr. Abraham Jacobi reports two cases of diphtheria of the external ear, one of which had (the other had not) been the seat of what he calls "catarrhal" inflammation. He cites a case of primary diphtheria of the external auditory canal, reported by Wreden, and refers also to several other cases already reported above.

Dr. Charles H. Burnett reports a case where, in a "child sixteen months old, without any previous symptoms of pain or acute inflammation in the ear, a large, cold abscess formed behind the auricle, pus ran from the meatus, the abcscess was opened by the family's medical adviser, and denuded bone was found extending along the posterior wall of the mastoid portion." This was part of the diphtheritic disease from which the child was at the time suffering.

I give here titles of two articles upon this subject, of which I could not at present obtain a copy or abstract:

Dr. G. Cerrutti: Otite externa pseudomembranosa.

Dr. J. Boke: Kroupose Entzündung des aussern Gehororganes, etc.

The primary form of diphtheritic otitis externa, if properly treated, is not apt to be widely destructive. It is characterized by its painfulness, and the production of the diphtheritic membrane.

On the contrary, the secondary form, or one following diphtheria of the fauces and middle ear, is very apt to be widely destructive, ofttimes being attended by great necrosis. It is characterized by its comparative painlessness and rapid transition into the chronic state.

Treatment, constitutional and local, must be supporting, antiphlogistic, antiseptic, and cleansing, modified and adapted to the individual case under attention.

Diphtheria of the middle ear, and middle ear tract, is by far more frequent than one would reasonably expect from the number of so-called "cures" of diphtheria. Of the disease in this region, Dr. Adam Politzer says that the occurrence has been established by the reported observations of Drs. Wendt, Wreden, Moos, Bezold, Kupper, Burkhardt-Merian, Gottstein, Blair and others. The primary form of diphtheria of the middle ear is supposed to have been demonstrated in two cases only, which have been reported by Dr. Burkhardt-Merian.

Post-mortems show the extension of diphtheria from the naso-pharynx to the tympanum.

Dr. Wendt, in his cases of naso-pharyngeal diphtheria, reports that 40 per cent. had the same process in the tympanum.

Dr. Wreden reports eighteen cases of the same.

In his discussion of the etiology of acute purulent otitis media, Dr. A. Politzer speaks of diphtheria as a cause, and says: "A consecutive otitis media takes place very often in scarlatinal naso-pharyngeal diphtheritis, as I have been informed by Viennese surgeons in most extensive practice among children."

Again, in discussing the etiology of chronic purulent otitis media, he says: "The suppurations of the middle ear, however, which most frequently become chronic, are those which arise in the course of typhus or the acute exanthemata, and the scarlatinal and diphtheritic forms are specially characterized by their obstinate course, which frequently defies all treatment."

Dr. D. B. St. John Roosa mentions the fact that diphtheria plays an important part among the factors of acute aural disease, and says that the suppurative forms is the one usually recognized.

Dr. Albert H. Buck reports a case of apparent diphtheritic inflammation of the middle ear.

Dr. J. C. Mulhall reports a case of invasion of the tympanum by diphtheria of the naso-pharynx.

In a report of over two thousand consecutive cases of ear diseases treated by our division of the Aural Staff of the New York Eye and Ear Infirmary, to which is appended a working schedule —a nomenclature and classification of diseases of the ear, you will find mentioned croupous inflammation of the external auditory canal, croupous and diphtheritic otitis media, diphtheria as a complication of disease of the middle ear tract, croupous and diphtheritic inflammation of the Eustachian tube, complicated with this the disease in the naso-pharynx, and paresis of the muscles of the Eustachian tube.

Drs. A. Jacobi, A. Politzer, C. H. Burnett, and others too numerous to mention, emphasize the frequent invasion of the middle ear by naso-pharyngeal diphtheria.

References might be multiplied if the occasion permitted, or necessity demanded; but these will suffice to place you upon your guard while attending cases of diphtheria.

Let me sum up by reminding you that aural invasion by diphtheria is very insidious, usually comparatively painless, showing a marked tendency uniformly to assume the chronic form, and apt to produce very rapid and wide-spread destruction, with necrosis, and burrowing to neighboring parts. Among its probable effects may be expected one of the following: fetid discharges, often persistent, always annoying and disgusting, deafness, erosion of the Eustachian tube, mastoid perforation, necrosis, partial or facial paralysis (Bell's Palsy), meningitis, thrombosis, embolism, phlebitis, pyæmia, cerebral abscess and death. These dangers, in view of the insidious development of aural diphtheria, should lead you to examine the ear early and frequently in every case of diphtheria which comes under your supervision. Should you find an inflamed and bulging drumhead, even if the patient make no complaint of aural discomfort or pain, it is your duty to make an incision of the drumhead—not a "pinhole," but an opening—sufficient to permit the escape of the products of inflammation, removing them by artificial means, if necessary, to check the burrowing to neighboring regions.

Let the practice of syringing the fauces and nares—which you have heard vigorously recommended for cases of diphtheria with prominent naso-pharyngeal manifestations—be prudent and careful, lest you force materies morbi within the Eustachian tube or into the middle ear. Whatever or wherever the local phenomena, pay close attention to the constitutional condition; treat your *patient;* and in modifying and adapting local measures for management of an aural development of diphtheria, let free drainage, cleanliness and antisepsis be your aim.

In closing, I recommend to you personal consultation of the literature of otology on the subject of diphtheria, so that you may further familiarize yourselves with the facts, and, having done so, act the more confidently and intelligently for the protection of your helpless but trustful patients.—*Weekly Medical Review.*

